# RETRACTED: Sobeh et al. A Polyphenol-Rich Fraction from *Eugenia uniflora* Exhibits Antioxidant and Hepatoprotective Activities In Vivo. *Pharmaceuticals* 2020, *13*, 84

**DOI:** 10.3390/ph19071038

**Published:** 2026-07-03

**Authors:** Mansour Sobeh, Marwa S. Hamza, Mohamed L. Ashour, Mona Elkhatieb, Mohamed A El Raey, Ashraf B. Abdel-Naim, Michael Wink

**Affiliations:** 1 AgroBioSciences Research Division, Mohammed VI Polytechnic University, Lot 660–Hay MoulayRachid, Ben-Guerir 43150, Morocco; 2 Institute of Pharmacy and Molecular Biotechnology, Heidelberg University, 69120 Heidelberg, Germany; 3 Department of Clinical Pharmacy Practice, Faculty of Pharmacy, The British University in Egypt, El-Sherouk City, P.O. Box 43, Cairo 11837, Egypt; marwa.hamza@bue.edu.eg; 4 Department of Pharmacognosy, Faculty of Pharmacy, Ain Shams University, Cairo 11566, Egypt; ashour@pharma.asu.edu.eg; 5 Department of Biochemistry, Faculty of Pharmacy and Biotechnology, German University in Cairo, Cairo 11835, Egypt; mona.el-khatieb@guc.edu.eg; 6 Department of Phytochemistry and Plant Systematics, National Research Center, Dokki, Cairo 12622, Egypt; elraiy@gmail.com; 7 Department of Pharmacology and Toxicology, Faculty of Pharmacy, King Abdulaziz University, P.O. Box 80260, Jeddah 21589, Saudi Arabia; abnaim@yahoo.com

The journal retracts the article entitled “A Polyphenol-Rich Fraction from Eugenia uniflora Exhibits Antioxidant and Hepatoprotective Activities In Vivo” [1], cited above.

Following publication, concerns were brought to the attention of the Editorial Office regarding the origins of the presented data, the accuracy of its methodological information, and the ethical oversight of the study presented in this article [1].

In accordance with the journal’s procedures, the Editorial Office and Editorial Board conducted an investigation which identified indications of duplication between the control panels of Figure 5 published in [1], Figure 5 published in earlier publications [2,3], and Figure 4 published in [4], representing different experimental conditions produced by the similar author group. Additional concerns regarding the accuracy of methodological information and lack of detail in supporting ethical documentation undermined the confidence of ethical oversight. Although the authors fully cooperated with the investigation, the explanations and supporting materials provided were not deemed sufficient by the Editorial Board to resolve the identified concerns. As a result, the Editorial Board has lost confidence in the reliability of the findings reported in this article and has decided to retract the publication as per MDPI’s retraction policy.

This retraction was approved by the Editor-in-Chief of the journal *Pharmaceuticals*.

The authors have been informed of this retraction and have indicated their disagreement with this decision.

The sixth author (A.B. Abdel-Naim) informed the journal that he was responsible for performing the hepatoprotective experiments and other contributions (e.g., image validation) and apologized for this oversight.
